# Topoisomerase 2 Alpha Cooperates with Androgen Receptor to Contribute to Prostate Cancer Progression

**DOI:** 10.1371/journal.pone.0142327

**Published:** 2015-11-11

**Authors:** J. L. Schaefer-Klein, Stephen J. Murphy, Sarah H. Johnson, George Vasmatzis, Irina V. Kovtun

**Affiliations:** 1 Department of Molecular Medicine, Mayo Clinic, Rochester, Minnesota, United States of America; 2 Department of Molecular Pharmacology and Experimental Therapeutics, Mayo Clinic, Rochester, Minnesota, United States of America; 3 Center for Individualized Medicine, Mayo Clinic, Rochester, Minnesota, United States of America; II Università di Napoli, ITALY

## Abstract

Overexpression of TOP2A is associated with risk of systemic progression in prostate cancer patients, and higher levels of TOP2A were found in hormone-resistant cases. To elucidate the mechanism by which high levels of TOP2A contribute to tumor progression we generated TOP2A overexpressing prostate cancer cell lines. We show that TOP2A promotes tumor aggressiveness by inducing chromosomal rearrangements of genes that contribute to a more invasive phenotype. Anti-androgen treatment alone was ineffective in killing TOP2A overexpressing cells due to activation of an androgen receptor network. TOP2A poisons killed tumor cells more efficiently early in the progression course, while at later stages they provided greater benefit when combined with anti-androgen therapy. Mechanistically, we find that TOP2A enhances androgen signaling by facilitating transcription of androgen responsive genes, thereby promoting tumor cell growth. These studies revealed a relationship between TOP2A and androgen receptor signaling pathway that contributes to prostate cancer progression and confers sensitivity to treatments.

## Introduction

DNA topoisomerase 2 alpha (TOP2A) is an essential nuclear enzyme, required for resolution of topological stress associated with DNA replication. TOP2A introduces transient double strand breaks (DSB) on DNA in an ATP-dependent fashion to allow changes in DNA topology and eliminate over-winding [1,2]. TOP2A function is crucial in many biological processes, including replication, transcription, DNA repair and chromosome structure maintenance. It is expressed at high levels in dividing cells as its level is known to be regulated through cell cycle, and it is often used as a proliferative marker [3,4]. Abnormalities of TOP2A protein are linked to chromosomal instability [5]. TOP2A is believed to play a major role in the decatenation checkpoint during mitosis, a mechanism responsible for correct chromosomal segregation [6–8]. High level of TOP2A as an indicator of more aggressive behavior and advanced stage was reported for several cancers [9,10]. Targeting TOP2A and subsequent cell proliferation, has been used as a therapeutic approach to routinely treat some malignancies [11]. Drugs belonging to the class of TOP2A poisons make up most of the approved for clinical use agents. Etoposide and doxorubicin target both TOP2 enzyme isoforms, A and B, by interfering with their DNA cleavage/ligation cycle, inducing DNA DSBs and trigger cell death [12,13]. A second class of TOP2 targeting compounds, TOP2 catalytic inhibitors, do not induce formation of protein-linked DNA DSB but act as non-competitive inhibitors of TOP2 ATPase activity [12]. The efficacy of TOP2A poisons is believed to depend on the level of TOP2A protein [5] and its enzymatic activity [14]^.^ A number of studies have shown that elevated levels of TOP2A account for higher sensitivity of the cells to TOP2A poisons etoposide and doxorubicin [15,16]. Activity of the TOP2A enzyme is regulated by post-translational modifications [14–22]. Phosphorylation of TOP2A at residues within the catalytic domain affects its enzymatic activity [17, 21]; and mutations at some of these sites were reported to account for resistance of tumor cells to TOP2A poisons [14]. Although, levels of TOP2A protein and its activity are considered major contributors to sensitivity of tumor cells to TOP2A poisons, TOP2A has also been shown to associate with resistance to chemotherapy, both *in vitro* and *in vivo*, through mechanisms of alteration of intracellular distribution and apoptosis inhibition [23,24]. High levels of protein DLX4 were shown to stimulate repair of DNA DSB induced by doxorubicin rendering the drug inefficient [25].

Resistance to TOP2A poisons, observed in clinically refractory tumors [26], and development of secondary cancers, such as myelogenous leukemia, as a result of *de novo* rearrangements [12,27], are the major limitations of anti-TOP2A therapy. Further investigation to fully understand factors and mechanisms underlying tumor response to TOP2A inhibiting drugs is needed.

TOP2A is frequently overexpressed in aggressive prostate cancer (PCa). Earlier studies have shown a positive correlation between expression level of TOP2A and Gleason score (GS) [28,29]. The carcinomas with the highest expression of TOP2A were poorly differentiated [28]. Elevated levels of TOP2A were also found in hormone-resistant PCa of GS 8–10 [29]. Our group has found that overexpression of TOP2A was significantly associated with increased risk of systemic progression in PCa patients [30]. The TOP2A protein level was the strongest predictor of outcome in the context of *ERG* expression [31]. *TMPRSS2-ERG* fusion, the most common rearrangement observed in PCa affects up to 60% of the cases [32,33]. The mechanism underlying generation of *ERG* fusion has been examined [34,35]. Haffner et al. have shown that androgen receptor (AR) and TOP2B are co-recruited to regulatory elements of androgen responsive genes upon transcription and trigger formation of DNA DSB. These breaks are believed to be highly recombinogenic and when repaired result in production of *de novo* fusion genes, such as *TMPRSS2-ERG* [35]. Similarly, TOP2B is recruited to other steroid receptors: estrogen receptor target genes upon estrogen signaling [36,37]. Although, TOP2A and B proteins have similar cellular functions in resolving DNA overwinding by introducing DSBs, no study reported a cooperation of TOP2A with transcription machinery in a fashion analogous to TOP2B. Despite a reported correlation between high levels of TOP2A and poor outcome in cancer, the exact mechanism underlying more aggressive phenotype associated with TOP2A is not known. TOP2 poisons, such as mitoxantrone and doxorubicin, are occasionally prescribed to treat castration-resistant metastatic PCa were shown to provide only palliative benefits [38]. On the other hand, standard adjuvant androgen deprivation therapy appeared to be effective only in the group of patients whose tumors expressed high level of TOP2A and are positive for *ERG* fusion [30,31]. It remains unclear whether these patients can benefit from therapy with TOP2A inhibitors in an adjuvant setting. In this study, we developed a cell model to examine role of TOP2A in tumor progression and assess its contribution to cell sensitivity to hormonal and chemotherapy. We show that TOP2A drives PCa towards a more invasive phenotype by inducing DNA rearrangements. We also provide evidence for the mechanism of time-related sensitivity of prostate tumor cells to TOP2A poisons. We demonstrate that more advanced prostate tumor cells, resistant to androgen ablation, can be more efficiently eliminated by a combination therapy with anti-androgen and doxorubicin. TOP2A interferes with anti-androgen therapy increasing sensitivity to AR signaling by facilitating its transcriptional activity at the androgen responsive elements.

## Results

### Generation of prostate cancer cell lines overexpressing TOP2A

We hypothesized that high levels of TOP2A, beyond those normally present in cycling cells, cause persistent generation of DNA DSB which subsequently lead to genomic rearrangements associated with formation of a more aggressive phenotype (S1A Fig). To investigate the contribution of TOP2A to prostate carcinogenesis and explore benefits of therapeutic targeting of this protein for PCa patients we set out to develop PCa cell lines stably overexpressing TOP2A. Although TOP2A is highly expressed in cancer cells *in vivo*, attempts to ectopically express this protein *in vitro* have been unsuccessful because of concomitant induction of apoptosis [39]. *In vivo*, during cancer progression, cells have evolved mechanisms to circumvent programmed cell death associated with high level of TOP2A. For example, CKS2 protein, elevated in PCa, in animal models and PCa cell lines, was shown to protect cells from apoptosis [40]. Thus, to overcome apoptosis induced by high levels of TOP2A, we first generated PCa LNCaP stable clones overexpressing CKS2 (S1B Fig), and clones with reduced level of caspase-3 (S1C Fig), a major effector caspase in apoptotic pathway [41]. Expression of caspase-3 was reported lost or reduced in PCa [42]. The catalytically inactive forms of caspase-3, when transfected into the cells, were specifically shown to inhibit cell death triggered by DNA damage response [43]. Attempts to circumvent TOP2A-induced apoptosis by overexpressing CKS2 failed to rescue any TOP2A overexpressing cell lines. Three clones with reduced level of caspase-3 were selected upon integration of TOP2A cDNA and used in subsequent experiments. T14 clone showed the highest level of TOP2A protein and was used as TOP2A high expressor, while T25 and T33 had an endogenous level of TOP2A and were used as negative controls (S1D Fig).

### Higher levels of TOP2A increase invasiveness of tumor cells

**Fig 1:**
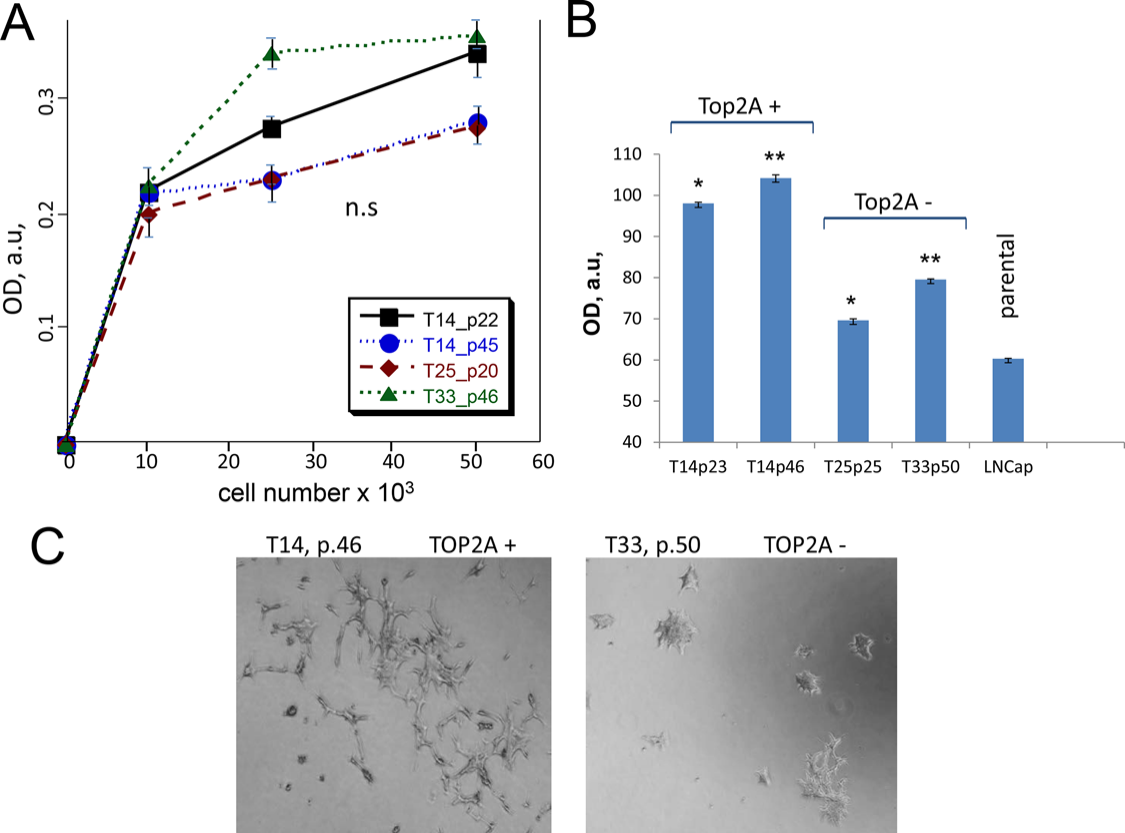
TOP2A promotes aggressive invasive phenotype. **A)** Proliferation of clones stably overexpressing TOP2A (T14) and matched control cells with endogenous level of TOP2A (T25 and T33). Proliferation was determined at 96 hours after cell plating. **B)** Motility of TOP2A clones of different passage and parental LNCaP cells determined using Boyden chamber assay. **C)** Images of cells with invasive phenotype after 72 hours of proliferation. P designates passage number; OD, au is measured optic density in arbitrary units. Data are presented as mean ± SD, based on 3 independent experiments. *p < 0.00001, and **p < 0.0001, n = 3.

To recapitulate changes that occur *in vivo* upon PCa progression, we continuously cultured clones to investigate how level of TOP2A affected cellular functions. Clones were harvested at different passages and analyzed. As expression of TOP2A is known to correlate with proliferation [4] we first examined proliferation rates. No significant difference in proliferation rates was found between the TOP2A higher expresser clone (T14) and lower expresser clones (T33 and T25) (Fig 1A). Proliferation rates did not change significantly with increasing passage of either clone suggesting that elevated levels of TOP2A do not affect tumor growth *per se* in culture. Higher levels of TOP2A did, however, affect motility and invasiveness of LNCaP clones. Parental LNCaP cells, TOP2A high and low expressers of early and late passages were compared in Boyden chamber assays. Both early (p23) and late (p46) passages of the TOP2A overexpressing T14 clone demonstrated greater movement through the membrane than their matched control clones or parental LNCaP cells (Fig 1B). Similarly, cells with high level of TOP2A demonstrated more invasive properties than their counterparts with endogenous level of the protein. The morphology of the cells migrated through the coated membrane differed between T14 and T33 clones. The TOP2A overexpressing T14 cells were spreading with irregular spiky shapes and longer processes resembling mesenchymal phenotype, whereas, the T33 cells appeared flatter and rounder, with short processes (Fig 1C).

### Sensitivity of cells overexpressing TOP2A to treatments changes upon prolonged proliferation

**Fig 2:**
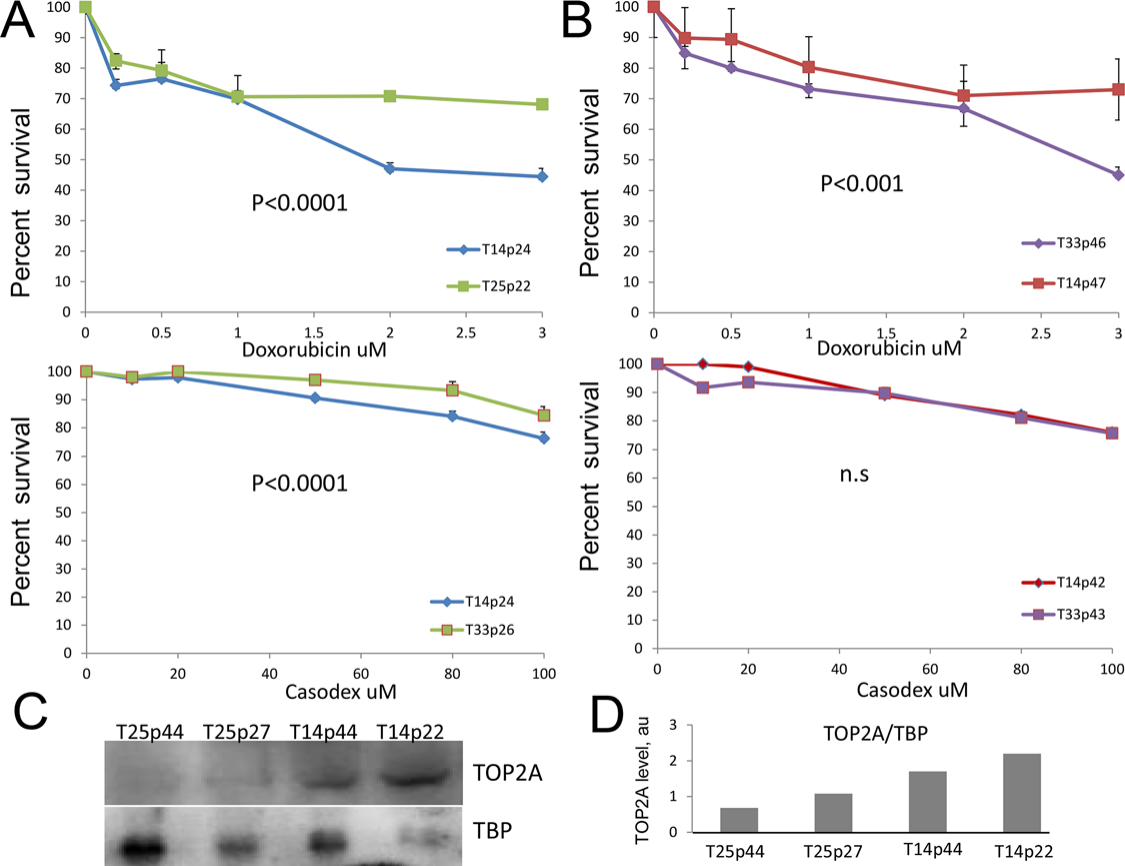
Sensitivity of TOP2A overexpressing to treatment with TOP2A poison and anti-androgen. **A)** Survival of TOP2A clones of early passage in response to treatment with doxorubicin (top panel) and casodex (bottom panel). **B)** Survival of TOP2A clones of late passage in response to treatment with doxorubicin (top panel) and casodex (bottom panel). **C)** Western blot showing levels of TOP2A in generated stable clones of early and late passages. Detection of nuclear protein TBP was used to verify equal loading. **D)** Quantification of TOP2A expression based on Western blot in C. Data are presented as mean ± SD, based on 3 independent experiments. P values are as indicated, n.s. is not significant.

High grade prostate tumors *in vivo* are known to develop resistance to androgen deprivation therapy with time [44]. No targeted treatment exists for these patients. We, therefore, investigated how sensitivity of generated TOP2A overexpressing clones to anti-androgens, TOP2A poisons and combination of two drugs changed with time. At early passages high TOP2A-expressor T14 cells were much more sensitive to doxorubicin than their counterpart T25 cells (Fig 2A). The difference disappeared at higher passages as cells were propagated in culture, with T14 cells becoming more resistant to the anti-TOP2A treatment (Fig 2B). Similar trend was observed for treatments with anti-androgen casodex (Fig 2A and 2B, bottom panels). There was a consistent significant difference in sensitivity to casodex between T14 and T25 clones of early passages. Although small (approximately 10–15%) at the selected doses, this difference was not observed at the higher passages, similar to pattern of sensitivity shown for doxorubicin (Fig 2B, top panel). The differences in response were not related to changes in the level of TOP2A protein as these cells after propagation in culture retained higher levels of TOP2A (Fig 2C). Thus, these data show that cells of early passages overexpressing TOP2A are more sensitive to treatments, both anti-TOP2A drugs and anti-androgens. A recent study has identified that DLX4, frequently overexpressed in breast and ovarian cancers, stimulates repair of DNA DSB induced by TOP2A poisons, thereby decreasing cell sensitivity to the treatment [25]. We therefore, compared level of DLX4 in normal prostate epithelial cells and tumor cells from cancers of different GS (S2 Fig). Microarray expression analysis [30] showed that there is no induction of DLX4 in PCa, thus indicating that DLX4 will not be a contributing factor to resistance to TOP2A poisons in PCa.

**Fig 3:**
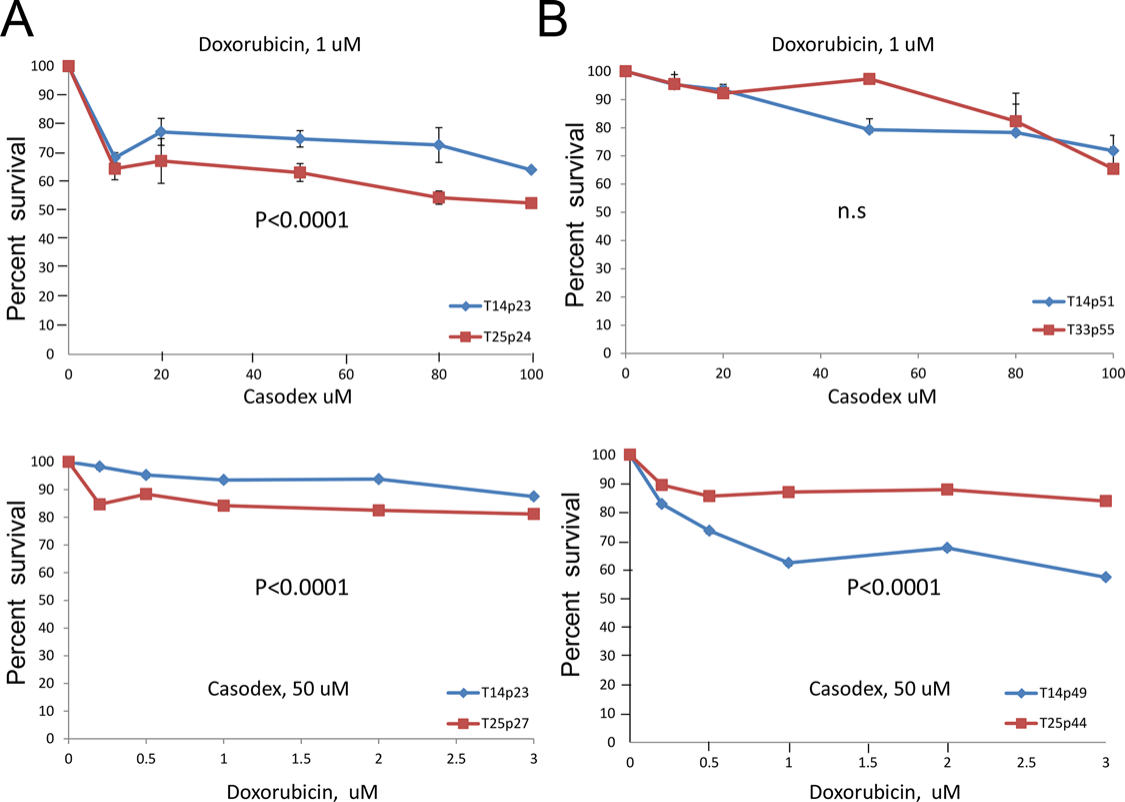
Sensitivity of TOP2A overexpressing cells to combination treatment with TOP2A poison and anti-androgen. **A)** Survival of TOP2A clones of early passage in response to combination treatment: top panel- constant dose of doxorubicin and increasing concentrations of casodex; bottom panel-constant dose of casodex and increasing concentrations of doxorubicin. **B)** Survival of TOP2A clones of late passage in response to combination treatment, drugs and concentrations are as in a. Data are presented as mean ± SD, based on 3 independent experiments. P values are as indicated, n.s. is not significant.

We next tested whether the cell killing would be more efficient if a combination of these two drugs was applied. In the first set, treatment with constant dose of doxorubicin and increasing concentrations of casodex showed no advantage in killing cells with high level of TOP2A as compared to endogenous control (Fig 3A and 3B, top panels). In fact, early passage T14 clone showed more resistance to this regiment compared to T25 (Fig 3A, top panel). In contrast, treatment with constant dose of 50 uM of casodex, application of which alone resulted in 90% cell survival (Fig 2), showed more efficient killing of late passage cells overexpressing TOP2A compared to the early passage and low expressing control T33 clone (Fig 3B, bottom panel). Collectively, these results suggest that combination treatment targeting AR and TOP2A in TOP2A highly expressing cells may be beneficial for advanced tumors.

**Fig 4:**
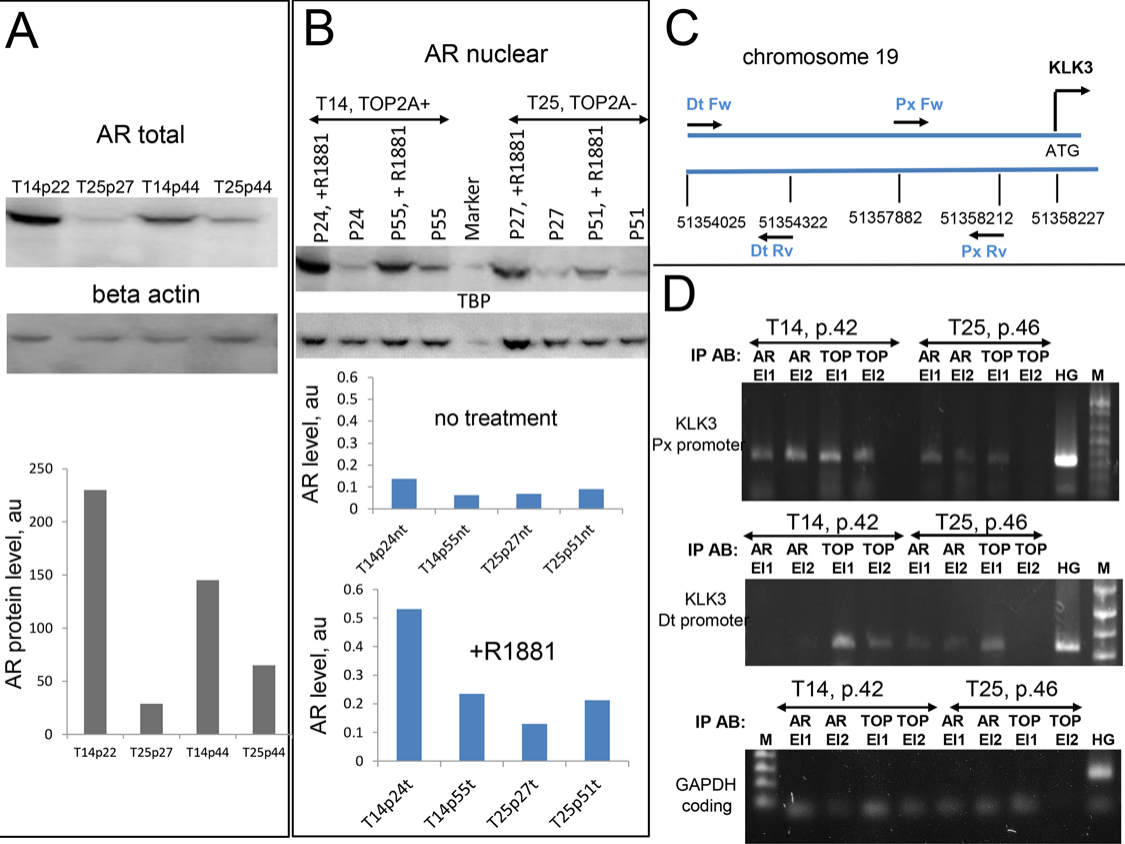
TOP2A protein is co-recruited to promoters of androgen responsive elements to stimulate gene expression. Western blot showing total level of AR in TOP2A clones of different passages (as indicated), top panel. Quantification of AR expression normalized to beta actin level, bottom panel. **B)** Western blot showing induction of AR level (top) upon treatment of TOP2A clones.with 5 nM of R1881. Corresponding quantification of TOP2A levels normalized to TBP loading control is shown (bottom); nt is non-treated, t is treated; cell passages are as indicated. **C)** Schematic showing promoter area for *KLK3* (PSA) gene used in CHIP assay. DtFw and DtRv depict positions of forward and reverse primers respectively for distal promoter region; PxFw and PxRv -positions for forward and reverse primers of proximal promoter. **D)** CHIP analysis of TOP2A clones stimulated with 5 nM of R1881 for 3 hours. Enrichment of AR and TOP2A at distal and proximal promoter regions of KLK3 gene (top and middle panels) and their absence at coding region of GAPDH gene (bottom panel) are shown. M is DNA size marker, HG-is human genomic DNA used as a positive control for PCR amplification, El1 and El2 are consecutive elution fractions (buffers are described in Methods).

To gain insight into possible interplay between TOP2A actions and androgen signaling network we next compared levels of AR in clones overexpressing TOP2A to those in clones with endogenous level of TOP2A. Cells were grown in media with non-stripped serum containing base level of androgens without additional supplementation. The total AR protein levels were compared between T14 and T25 clones of early and late passages (Fig 4A). T14 cells of both passages showed significantly higher levels of AR than the matching control T25 cells (elevated 3 and 8 fold respectively) suggesting an involvement of TOP2A in induction of AR expression at the transcription level. The levels of TOP2A protein remained higher in T14 cells at each passage taken into experiment (Fig 2C, S3C and S3E Fig). To ensure that TOP2A protein was functional, the assay using TOP2A specific DNA template was performed. Enzymatic activity measured in protein extract obtained from T14 cells was higher than that of T25 cells (S3 Fig). When adjusted to the amount of total protein, it was 6 fold greater (S3B Fig), consistent with higher levels of TOP2A in T14 compared to T25 cells. Total enzymatic activity remained elevated, consistent with higher levels of TOP2A protein, in later passages of T14 cells (S3D Fig), confirming that TOP2A remained functional, and there were no mutations in active site introduced upon propagation of this clone.

### TOP2A is modulating AR signaling

Higher levels of AR were observed in TOP2A overexpressing cells (Fig 4A). To elucidate the mechanism by which TOP2A may contribute to regulation of transcription, we treated cells with synthetic androgen R1881 and compared nuclear levels of AR. AR translocates to the nucleus upon ligand binding to regulate transcription of responsive genes [45]. We observed significantly higher level of nuclear AR in response to androgen stimulation in TOP2A overexpressing T14 cells of early (p.24) passage compared to T25 cells (Fig 4B). The level of nuclear AR in these cells increased 5 fold after the treatment. Late passage (p.55) cells overexpressing TOP2A showed a slightly elevated level of AR, not as dramatic as their precursors at passage 24. Activated AR recognizes and binds to palindromic androgen response element (ARE) sequences at the proximal genes to induce their transcription [45,46]. Our data suggest that TOP2A acts with AR to activate transcription of responsive genes. To formally test this we performed chromatin immunoprecipitation using a region corresponding to the proximal and distal ARE in the promoter of prostate-specific antigen (PSA) (KLK3 gene, Fig 4C), a well characterized transcriptional target of AR [46]. Antibodies, to TOP2A and AR, were able to capture DNA fragments containing ARE at KLK3 promoter after treatment with R1881 (Fig 4D). There was no difference in binding of TOP2A or AR to AREs between T14 and T25 clones. The binding was specific as no recruitment of either TOP2A or AR to unrelated sequence was observed (coding region of GAPDH gene was used as a control. This result indicates that as does its homologue TOP2B, TOP2A is recruited to steroid regulatory elements to stimulate transcription and suggests synergistic actions with androgen signaling in prostate tumor cells.

### TOP2A promotes production of DNA rearrangements in PCa cells

**Fig 5:**
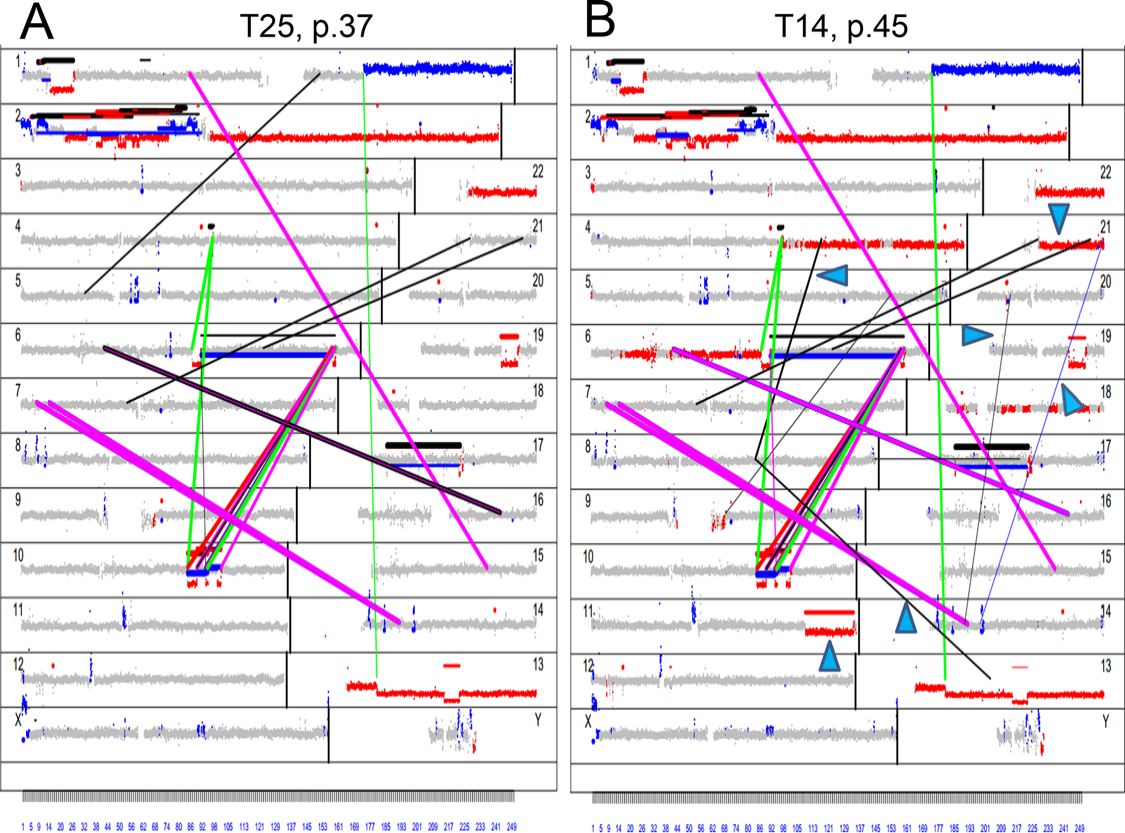
Genome plots showing landscape of DNA rearrangements in generated clones. **A)** Late passage of T25 clone expressing endogenous level of TOP2A. **B)** Late passage of clone T14 overexpressing TOP2A. Grey dots (counts) show frequency of distribution of reads in 30KB windows and breakpoints for all chromosomes (numbers are indicated). The X axis spans the length of the chromosome, the Y axis shows the number of reads for each window. Window counts are shown according to the prediction by CNV algorithm. Grey points are normal, red points correspond to deletions and blue points show gains. Lines connect bioinformatically identified breakpoints. The widths of the lines correlate with number of associated mate-pair reads. Color of the connecting lines indicates polarity of the joined chromosome. For intra-chromosomal events red shows forward direction for both pieces, green indicates inversion for one partner and blue shows inversion for both. For inter-chromosomal events, red connects the p-side piece from the larger chromosome to the q-side piece of the smaller chromosome in forward direction, green connects the q-side piece from the larger chromosome to the p-side piece of the smaller chromosome in forward direction, blue connects the p-side piece from the larger chromosome to the p-side piece of the smaller chromosome in reverse direction and magenta connects the q-side piece from the larger chromosome to the q-side piece of the smaller chromosome in reverse direction. Black indicates balanced translocations. Blue arrowheads point to selected DNA rearrangements that were acquired upon proliferation (absent in corresponding cells of earlier passage.

**Fig 6:**
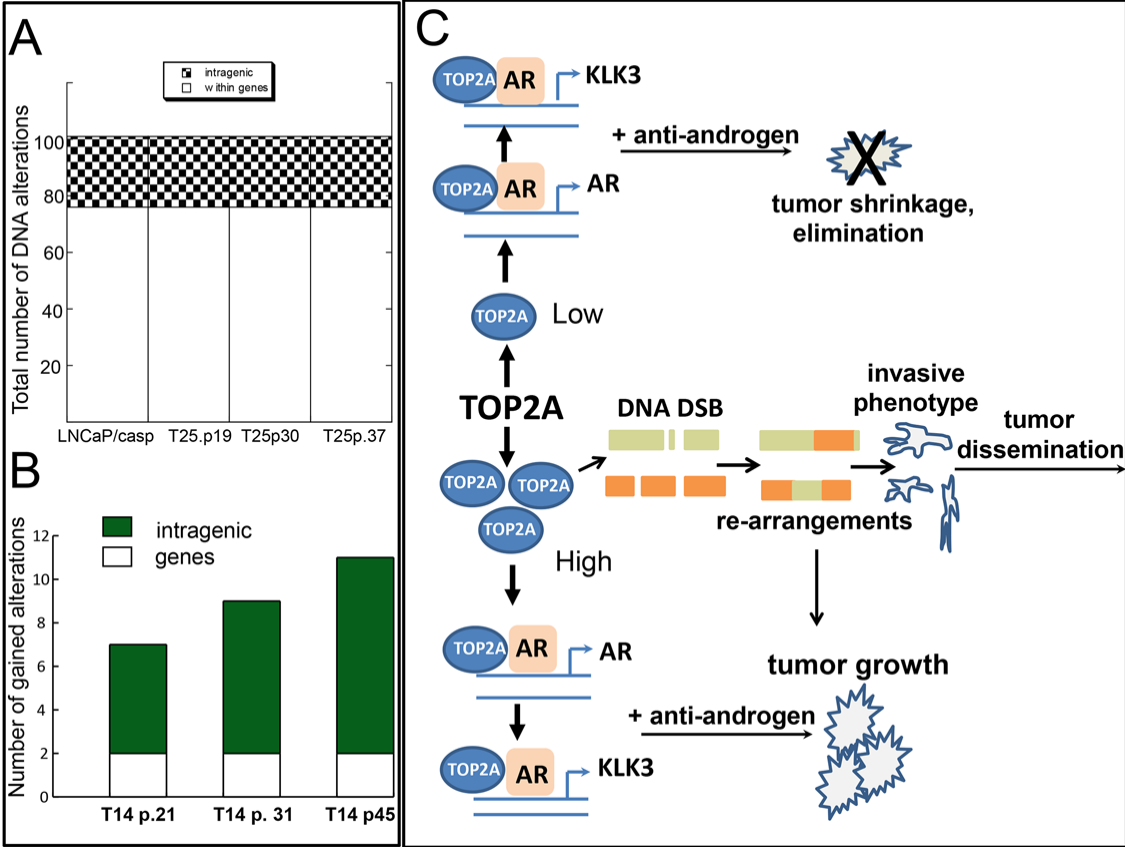
TOP2A promotes accumulation of DNA rearrangements in proliferation PCa cells. **A)** Total number of breakpoints present in T25 cells of different passages. LNCaP/casp is a derivative of LNCaP cell line stably expressing siRNA to knock down caspase 3. **B)** DNA alterations gained by TOP2A overexpressing T14 cells upon proliferation. Numbers represent alterations observed in addition to those present in control T25 clone. **C)** Schematic showing proposed role of TOP2A in prostate cancer progression and resistance to androgen ablation therapy.

Large chromosomal rearrangements are commonly observed in PCa [47,48] and are believed to play a role in tumor initiation and progression. We show here that in a fashion similar to TOP2B, TOP2A is recruited to AREs to stimulate transcription. Whether reiteration of this process results in progressive accumulation of DNA rearrangements was not investigated. To test this, mate pair breakpoint sequencing was performed [49,50] on different passages of high and low expressing TOP2A clones. Parental LNCaP cells harbor a number of genomic alterations that include translocations, and copy number changes (S4A Fig). The cells selected after integration of vectors carrying siRNA to caspase-3 and cDNA for TOP2A showed significantly higher number of chromosomal alterations (S4B Fig). This is not surprising since the manipulations likely led to selection of cells with reduced ability to undergo apoptosis. T25 clones expressing endogenous level of TOP2A did not show change in the number of genomic rearrangements with proliferation (Figs 5A and 6A). The total number of alterations in T25 cells of different passages was the same as in precursor cells harboring reduced level of caspase-3 (Fig 6A). In contrast, T14 cells overexpressing TOP2A demonstrated progressive accumulation of rearrangements with increasing passage (Figs 5B and 6B). The TOP2A gene itself was also affected in T14 of late passage (Table 1). However, the disruption of one allele did not result in significant decrease of expression of the protein, suggesting that exogenous integrated copy of the gene was mainly driving expression.

**Table 1:** Genes affected in TOP2A overexpressing clone.

Gene	Function	Type of alteration	Cancer observations
ANKS1B (EB1)	E2A-PBX1 nucleoplasmic coiling protein interactions	Duplication of parts	Elevated in pre-B cell 2
FOXO5 (Emi1)	Regulates progression through early mitosis	Duplication	Overexpressed in a number of cancers
PARD3B	Involved in asymmetrical cell division and cell polarization	Inversion, disruption of the gene	
TOP2A	Elimination of DNA overwinding	Inversion, translocation disruption of the gene	Often overexpressed in cancer
Tango6	Transport and Golgi organization	Deletion of exons 11–17	Homologue TANGO is tumor suppressor, down regulated or lost in melanoma
TBC1D5	GTPase-activating protein for Rab family	Deletion of exon 2	Loss of copy number in breast cancer

Together, these results indicate that genomic changes induced by high levels of TOP2A lead to acquisition of more aggressive phenotype and may also contribute to developing resistance to single agent treatments.

## Discussion

Although radical prostatectomy cures most of GS 7–10 patients, the risk of systemic progression and death after surgery remains higher than 15%. A subset of these patients develop disseminated disease [44] and some, after initial improvement with androgen deprivation therapy, progress to castration resistant state [51]. Despite advances in treatment of patients with castration resistant disease [52, 53], achievement of long term survival remains an issue. Understanding of mechanisms underlying resistance to ablation is critical. One of the proposed mechanisms is development of increased sensitivity to low levels of androgens after ablation due to AR amplification or elevation of its expression [54,55]. Consistent with this, our study revealed that TOP2A, frequently overexpressed in PCa cases at high risk of progression, drives expression of AR. Our data suggest that androgen ablation therapy might be less effective in the presence of high level of TOP2A protein (Fig 6C). Further, treatment experiments support the notion that therapy using combination of anti-androgen and TOP2A poison provides a stronger benefit. We also show that targeting TOP2A with poison alone is efficient only at earlier stages, before TOP2A-driven DNA changes have occurred (Fig 6C). In agreement with these findings a recent study using a different model for PCa has identified TOP2A as a recurrence–specific tumor antigen [56]. Mouse prostate tumors when exposed to suboptimal immunotherapy evolved into a drastically different phenotype, showing high expression of TOP2A and response to anti-TOP2A treatment. At this stage of minimal residual disease, TOP2A-positive cells displayed stem cell-like phenotype and showed increased invasiveness [56], a property, characteristic of TOP2A-overexpressing cells in our model. In another study, using syngeneic murine model of spontaneous PCa metastasis [57] authors demonstrated that high levels of TOP2A correlated with high levels of methyltransferase Ezh2, and targeting of both proteins in combination resulted in efficient cell death.

Besides the level and functionality of TOP2A, cellular response to its inhibitors was also reported to depend on activity of other factors. DLX4, a homeobox gene, overexpressed in several cancers [58], was shown to reduce sensitivity of tumor cells to TOP2A poisons by stimulating repair of DSB by non-homologous end joining [25]. Our data indicate that this is not the case in PCa, as the level of DLX4 does not increase with the tumor grade but remains similar to that in normal epithelial cells (S2 Fig).

Collectively, these studies suggest that there is a time window in the course of PCa progression where anti-TOP2A therapy might be particularly effective. Whether TOP2A poisons/inhibitors or immunotherapy has to be the choice of treatment in clinic remains to be determined.

Finally, we show here that TOP2A cooperates with AR to induce transcription of target genes (Fig 6C). The level of AR itself is significantly higher in TOP2A overexpressing cells after stimulation with androgen agonist. Similar to its isoform TOP2B, TOP2A is co-recruited to ARE with AR, and is likely to activate transcription. Previously, TOP2B was also shown to induce DNA DSB at regulatory regions of estrogen and AR target genes [35,36]. Although, in our study we did not address this possibility directly, we did observe an increase in a number of DNA rearrangements in TOP2A overexpressing cells upon proliferation. Whether they are the result of faulty repair of DNA DSBs which occur at the transcriptional regulatory elements or a consequence of excess of TOP2A function during replication remains to be investigated further. We identified a few rearranged genes that we consider as “TOP2A dependent” (Table 1). Based on the prior observations related to function of these genes, the changes in properties of TOP2A overexpressing cells and their sensitivity to treatments can be attributed to the alterations at those DNA regions. For example, Emi1 (FBOX5) (Table 1) affected in T14 cells of the latest passage is a known regulator of mitotic progression [59,60]. It has been implicated in determining sensitivity to doxorubicin [61]. In that study depletion of Emi1 led to increase in sensitivity, consistent with this, our data suggest that duplication observed in T14 cells of later passage may account for a decrease of sensitivity to doxorubicin (Fig 2B). Disruption of PARD3B gene, shown to control tight junctions [62], might be responsible for increase in motility and invasion capacity of T14 cells. Reduction in expression or loss of Tango, a homologue of Tango6, disrupted in TOP2A overexpressing cells, was also previously shown to increase migration [63]. The function of Tango6 has not been examined yet, whether its loss may have similar effect on PCa cells’ properties requires further investigation. Future studies are needed to elaborate on exact functional contribution of acquired genomic changes.

## Materials and Methods

### Cell culture and treatments

Cell cultures were maintained following ATCC recommendations. LNCaP (ATCC) and LNCaP stable derivatives were grown in RPMI 1640 (Gibco, Life Technologies) culture supplemented with 100 units/ml of penicillin, 1 mg/ml of streptomycin, and 10% FBS. For androgen treatment experiments cells were cultured in the same medium but replacing 10% FBS with 5% charcoal-filtered FBS (One Shot, Life Technologies) for 48 hours prior to addition of 5nM R1881 (metribolone, RM10, TSZCHEM), and after that were incubated for an additional 48 hours. Cells were collected by scraping and protein was isolated (Nuclear Extract kit, Active Motif) for Western blot analysis. Doxorubicin and casodex were purchased from Sigma-Aldrich Inc.

### Generation of cells overexpressing TOP2A

Panel of six caspase 3 shRNAs cloned into pGIPZ letiviral vector were acquired from Open Biosystems (ID: V2LHS_15044–15049 and used for transfection into LNCaP cells. To overexpress CKS2, LNCaP cells were transfected with pWZL-Neo-Myr-DEST CKS2 expression vector (Addgene). The presence of integrated CKS2 plasmid was confirmed by PCR (Fig 1A). Selected clones with knockdown of caspase-3 (level confirmed by Western blotting, Fig 1) and those showing integration of CKS2 cDNA were used for stable integration of TOP2A cDNA (pReceiver lentiviral vector E0236, Genecopoeia).

To create TOP2A stable cell lines, viral supernatant was generated by co-transfection of 293T cells with 2 mg each of pGag/Pol, pVSV, and either control or TOP2A constructs. After 2 days, medium was collected and centrifuged at 5,000 x g followed by filtration through a 0.22-mm syringe nylon filter (Fisher Scientific). Polybrene (10U/mL;Sigma) was added to viral supernatant and caspase-3 shRNA/LNCap cells were then transduced with TOP2A viral supernatant for 24 hours. Clones were selected using 2ug/ml puromycin.

### MTS proliferation assay

MTS cell viability assay was performed following manufacturer instructions (Promega Corp.). Cells were plated in 24-well plates and absorbance values were obtained using a GloMax multi detection system (Promega).

### Cell migration assay

The migration of generated stable clones was measured using CytoSelect migration assay (Cell Biolab) according to manufacturer’s protocol. Briefly, medium containing serum was placed in the lower chamber while cells in serum free medium were added to the upper chamber separated by a membrane with or without coating, then incubated for 24 hours in a cell culture incubator. Following incubation, cells in the upper chamber were stained, extracted and measured on a GloMax Multi plate reader (Promega).

### TOP2A enzyme activity

Topoisomerase II assay kit (TG1001-2, TopoGEN) was used to measure decatenation activity of TOP2A on relaxed supercoiled DNA template in generated stable clones. Briefly, extracts were prepared from TOP2A overexpressing and control cells, combined with DNA and buffer and incubated at 37C for 30 minutes. Stop buffer was added to the reaction and an agarose gel was run on the reactions along with appropriate decatenated and linear DNA markers.

### Western blotting

NuPAGE Novex B-T minigels and Surelock gel and transfer apparatus, (Life Technologies) were used for protein electrophoresis and subsequent immunoblot transfer. Antibodies were as follows, anti-TOP2A, TBP and beta-actin were from Abcam (ab12318, ab51814 and ab6276, respectively), androgen receptor and caspase primary antibody were from Santa Cruz Biotechnology, Inc. (sc-816 and sc-7272, respectively), secondary HRP conjugated antibodies (anti-mouse cat.# 170–6516 and anti- rabbit cat. # 170–6515) were from BioRad Laboratories, 7272. Chemiluminescent substrate, SuperSignal West Pico, was purchased from Thermo scientific.

### Chromatin immunoprecipitation (CHIP)

For CHIP experiment cells were cultured in the medium containing 5% charcoal-filtered FBS (One Shot, Life Technologies) for 48 hours, treated with 5nM of R1881 (metribolone) for 3 hours and cross-linking was done using 10% formaldehyde. Cells were washed and lysed. TOP2A antibody (Abcam, ab12318) and androgen receptor antibody (Santa Cruz, sc-816) were coupled to dynabeads and cross-linked according to manufacturer protocol (Novex, 1004D). Incubation, washing, elution and protein/DNA reversal of crosslink were performed as previously described [64]. PCR was performed with specific primers (S2 Table) to proximal −250 to −39 bp and distal −4170 to −3978 bp from transcription site of KLK3 gene (PSA) to identify TOP2A and androgen receptor binding to these sites. Primers specific to coding region of GAPDH were used as a negative control.

### Mate pair (MP) whole genome sequencing and bioinformatics analysis

Cell lysis, DNA isolation, library construction and mate pair whole-genome sequencing was carried out as described previously [65]. Bioinformatics analyses of MP data was performed using a 32-bit binary indexing of MP reads were aligned as previously described [S5, 49]. Plots of the counts of read-pairs mapped in non-overlapping consecutive 30KB windows were generated to cover the entire genome. A masking operation using normal samples eliminated aberrant hit counts such as genomic regions not represented in the reference genome and regions with repetitive sequences. The mask was developed using independent normal DNA treated the same as the cancer samples. Analysis of copy number variation and filtering and masking methods for false positives are described in S1 Information.

### Statistical analysis

All data were presented as mean ± SD. The mean was the average of at least triplicate samples in each experiment. Each experiment was repeated at least three times. Both Student’s t-test and two-way ANOVA statistical tests were used to analyze the results. Differences were considered to be statistically significant at p < 0.05.

## Supporting Information

S1 Fig Generation of prostate cancer cell lines overexpressing TOP2A. (TIF)

S2 Fig Expression of TOP2A and DLX4 in prostate tumor samples. (TIF)

S3 Fig Overexpressed TOP2A remains enzymatically active upon propagation of cells. (TIF)

S4 Fig Genome plots of DNA rearrangements in LNCaP cell line. (TIF)

S1 Table Mate pair sequencing data. (XLSX)

S2 Table Primers used in CHIP assay. (DOCX)

S1 Information (DOCX)
