# Supplementary figures and images for: Topoisomerase 2 Alpha Cooperates with Androgen Receptor to Contribute to Prostate Cancer Progression
Source: PLoS One. 2015 Nov 11;10(11):e0142327. doi: 10.1371/journal.pone.0142327 (PMC4641711; doi:10.1371/journal.pone.0142327)

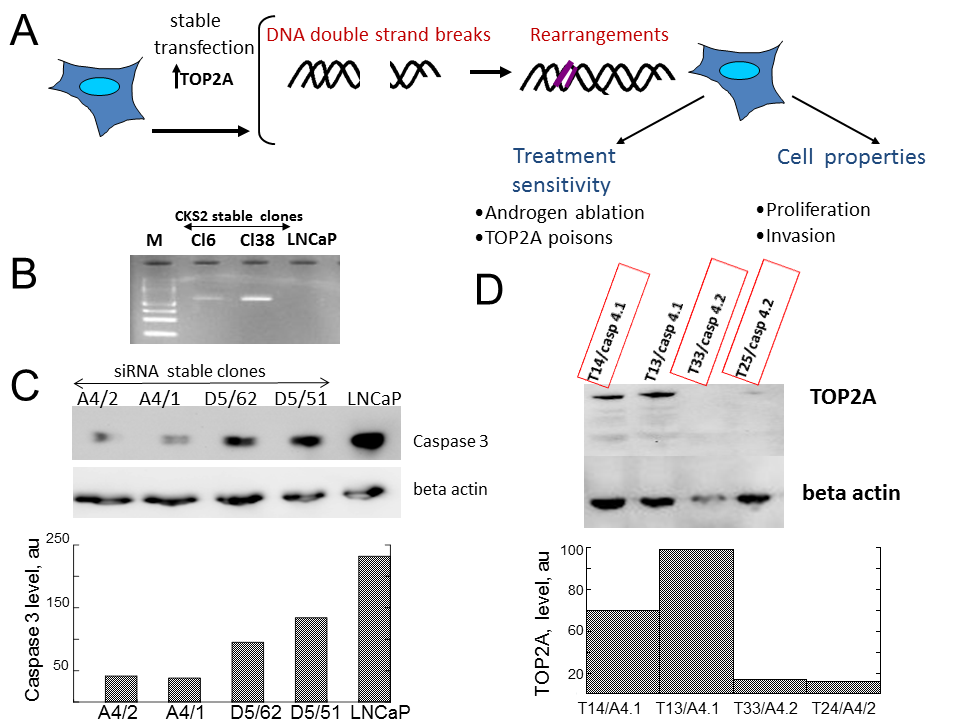

Supplement: S1 Fig — (TIF) [file pone.0142327.s001.tif]

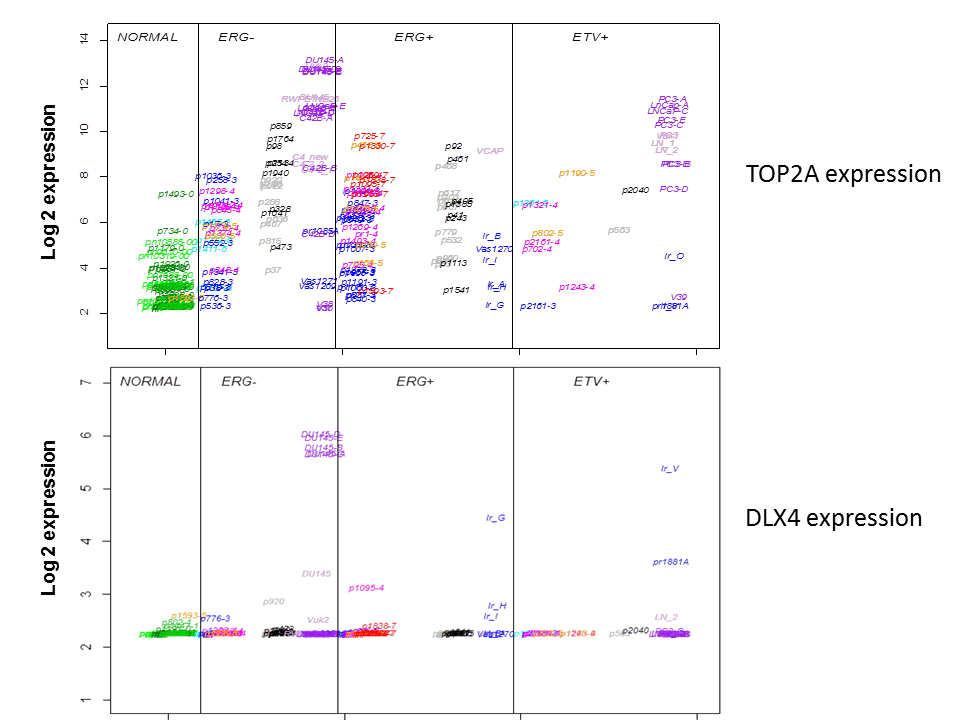

Supplement: S2 Fig — (TIF) [file pone.0142327.s002.tif]

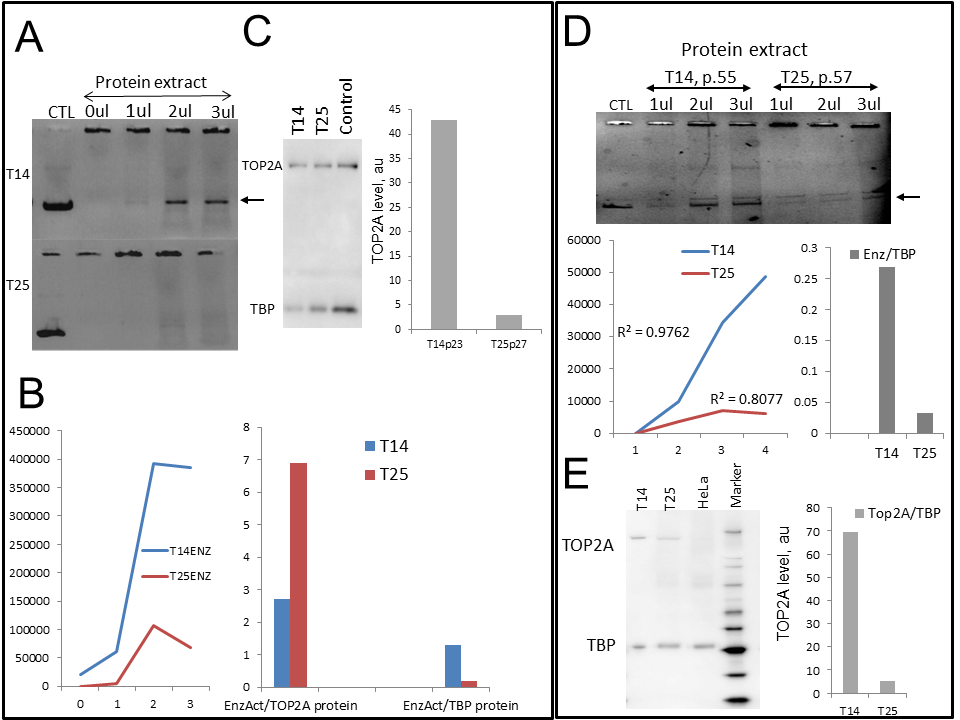

Supplement: S3 Fig — (TIF) [file pone.0142327.s003.tif]

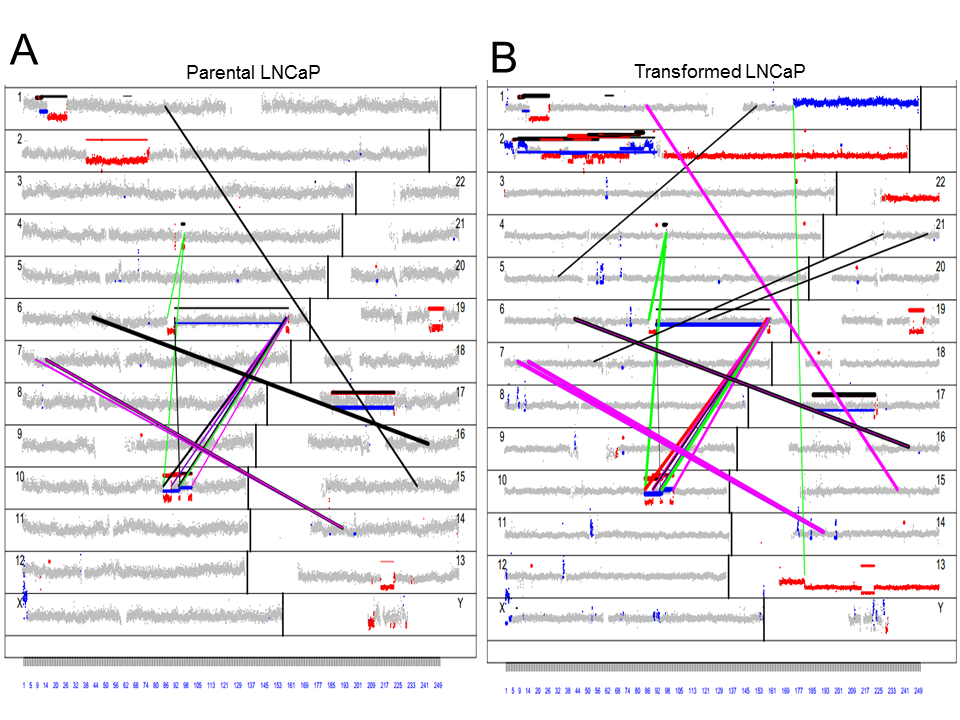

Supplement: S4 Fig — (TIF) [file pone.0142327.s004.tif]
