# Supplementary material for: Topoisomerase 2 Alpha Cooperates with Androgen Receptor to Contribute to Prostate Cancer Progression
Source: PLoS One. 2015 Nov 11;10(11):e0142327. doi: 10.1371/journal.pone.0142327 (PMC4641711; doi:10.1371/journal.pone.0142327)
Supplement: S2 Table — (DOCX) [file pone.0142327.s006.docx]

**Table S1.**  Primers used in CHIP assay

| PSA proximal promoter | Sequences |
| --- | --- |
| Forward 2 | GGATCGTACCCACCCCCTGTTT |
| Reverse 3 | CAACCGGGACCCACATGGTGA |
|  |  |
| PSA distal promoter |  |
| Forward 3 | CCTGCTCAGCCTTTGTCTCTG |
| Reverse 2 | GGGAGGCAATTCTCCATGG |
|  |  |
| GAPDH, coding region |  |
| Forward | ACAGTCCATGCCATCACTGC |
| Reverse 4 | GCAGGTTTTTCTAGACGGCAGG |
