# Supplementary material for: Topoisomerase 2 Alpha Cooperates with Androgen Receptor to Contribute to Prostate Cancer Progression
Source: PLoS One. 2015 Nov 11;10(11):e0142327. doi: 10.1371/journal.pone.0142327 (PMC4641711; doi:10.1371/journal.pone.0142327)
Supplement: S1 Information — (DOCX) [file pone.0142327.s007.docx]

**S7 Supporting Information**

**Methods**

Copy number variation (CNV). CNVs in MP data were identified by analyzing frequency distributions of window counts of mapped reads across the reference genome.  The analysis was based on the assumption that tumors have a dominant (primary) mode in their frequency distribution corresponding to normal diploid areas of the genome and minor (secondary) modes or outliers that correspond to copy number alterations.  The distribution of the primary mode was used to find windows with normal counts as well as windows with outlier counts.  Thresholds for copy number gains and losses were determined by analyzing lower and upper secondary modes. Since outcome is dependent on the size of the window, iterative sizing moving from large to small window was used to detect large variations more accurately. Window sizes varied from 30-to-3,000 Kb.  Counts were calculated for the tumor sample and also for the normal sample, experimentally treated the same as the tumor sample.  The correction vector from selected normal sample were then was used to normalize the count vector from the cancer sample to reduce the noise. In each iteration, a density distribution of the corrected counts was produced using the *density* function in R. To call deletions and amplifications peaks and valleys of the frequency distribution were determined by finding the spots where the discrete derivative of the distribution would cross zero.  The dominant mode was determined by finding the highest peak of that distribution which was also the maximum of the density function.  The nearest left minimum was considered a threshold below which deletions were called.  Amplifications were called by finding the neighboring minimum on the right of the highest peak.

Filtering and masking methods for false positives. Algorithmic filters were set to minimize the effects of both false-positive and false negative results. Namely, the lowest limit of MP associates to call an event was set at five, where the false-positive rate was practically zero; a mask of breakpoints was used to eliminate common variants and discordant mate pairs from experimental or algorithmic errors []. Five or more associated reads were required to be called a cluster. Occasional untrue clusters within this group were removed by a devised filtering step that took into consideration the abnormal spreads and overlaid mixed orientations of the reads.  Efficient algorithmic masks derived from the MP sequencing of 30 normal samples and over 1000 independent clinical tissues were used to eliminate the majority of the low level common variant events.

**S1 Fig. Generation of prostate cancer cell lines overexpressing TOP2A.**

**A)** Schematic showing design of experimental system to test TOP2A contribution to prostate cancer progression and sensitivity to treatments. **B)** Gel showing amplification product indicative of incorporation of CKS2 cDNA in the genome of LNCaP cells. M is a size marker.**C)** Western blot showing level of caspase-3 in generated siRNA knock down clones (top); bottom: plot showing normalized level of caspase-3. (D) Western blot showing level of TOP2A in clones stably transfected with TOP2A cDNA (top). Highlighted lanes correspond to clones which were selected for the study. Bottom: plot showing normalized level of TOP2A.

**S2 Fig.** **Expression of TOP2A and DLX4 in prostate tumor samples**.

Comparison of expression profiles of TOP2A (top) and DLX4 (bottom ) in prostate cancer samples. RNA for microarray analysis was extracted from laser microdiseccted tissue (30), adjacent Gleason pattern (GP) 3 and 4 of Gleason score 7 tumors were collected separately. Green corresponds to normal prostate epithelial cells, light blue is Prostatic intraepithelial neoplasia, dark blue is GP3, pink is GP4, orange is GP5, red corresponds to metastases, cell lines are shown in purple. Grey and black are bulk tissue (without laser capture microdissection) with good and bad (systemic progression) outcome respectively. Tumors are grouped into lacking *TMPRSS*-*ERG* fusion gene *(ERG-),* harboring *TMPRSS*-*ERG* fusion *gene (ERG+)* and harboring *ETV* fusion gene *(ETV+)*.

**S3 Fig. Overexpressed TOP2A remains enzymatically active upon propagation of cells.**

**A)** Representative gel showing decatenation activity of TOP2A in protein extracts of TOP2A clones of early passage. **B)** standard curves constructed based on the amounts of cleaved substrate. **C)** Western blot showing levels of TOP2A and TBP (loading control) in protein extracts (left panel) used for decatenation activity measurements in A, quantification of relative activity normalized to amount of TOP2A and TBP (right panel). **D)** Representative gel showing decatenation activity of TOP2A in protein extracts of TOP2A clones of late passage (top) and standard curves constructed based on the amounts of cleaved substrate (bottom). **E)** Western blot showing levels of TOP2A and TBP (loading control) in protein extracts (left panel) and quantification of relative activity normalized to amount of TOP2A and TBP (right panel). Arrows depict cleaved DNA product. CTL is control.

**S4 Fig. Genome plots of DNA rearrangements in LNCaP cell line.**

**A)** Genome plot showing landscape of DNA rearrangements in parental LNCaP cell line. **B)** Genome plot showing landscape of DNA rearrangements in LNCAP cells transformed with pGIPZ letiviral vector containing caspase-3 siRNA (Open Biosystems) and pReceiver lentiviral vector E0236contaning TOP2A cDNA (Genecopoeia)**.** The X axis spans the length of the chromosome, the Y axis shows the number of reads for each window. Designations are as in Figure 6.
